# Une occlusion intestinale aiguë par un os de poulet

**DOI:** 10.11604/pamj.2015.22.91.7932

**Published:** 2015-10-01

**Authors:** Ammar Mahmoudi, Mabrouk Abdelali

**Affiliations:** 1 Service de Chirurgie Générale et Digestive, CHU Fattouma Bourguiba de Monastir, Tunisie; 2 Service d'Imagerie Médicale, CHU Fattouma Bourguiba de Monastir, Tunisie

**Keywords:** occlusion intestinale aiguë, corps étranger, os de poulet, scanner, chirurgie, acute intestinal obstruction, foreign body, chicken bone, scanner, surgery

## Image en medicine

L'ingestion de corps étranger (CE) peut causer de multiples complications parfois graves. Il s'agit le plus souvent d'un accident survenant chez des enfants, parfois chez des malades psychiatriques. Chez les adultes, l'accident est favorisé par des mauvaises habitudes alimentaires (repas trop rapide, quintes de toux ou dispute lors du repas…). L'introduction des CE peut même être volontaire. La nature des CE ingérés par inattention dans le tube digestif est très variable. Le CE peut perforer le tube digestif et occasionner une péritonite, il peut même migrer hors du tube digestif et être à l'origine d'un tableau clinique inattendu (péricardite constrictive, perforation de la veine cave inférieure). Il peut être à l'origine d'une occlusion intestinale aiguë (OIA) par obstruction. Nous rapportons l'observation d'un homme âgé de 30 ans sans antécédents notables ayant présenté un syndrome occlusif depuis 24 heures. A l'examen, la température était à 37°C, l’état hémodynamique était stable. L'examen abdominal avait trouvé une distension abdominale, une sensibilité diffuse et un tympanisme péri-ombilical. Le scanner abdominal (A, B, C, D) avait montré une OIA de type grêlique en amont d'un obsatcle endoluminal calcifié au niveau de la valvule iléo-cæcale. Après aspiration gastrique et réanimation, le patient a été opéré par laparotomie médiane; il a été constaté une distension grêlique diffuse en amont d'un CE enclavé au niveau de la valvule iléo-cæcale (un os de poulet). Il a été réalisé une extraction du CE par une entérotomie et une vidange retrograde du grêle. Les suites opératoires étaient simples.

**Figure 1:**
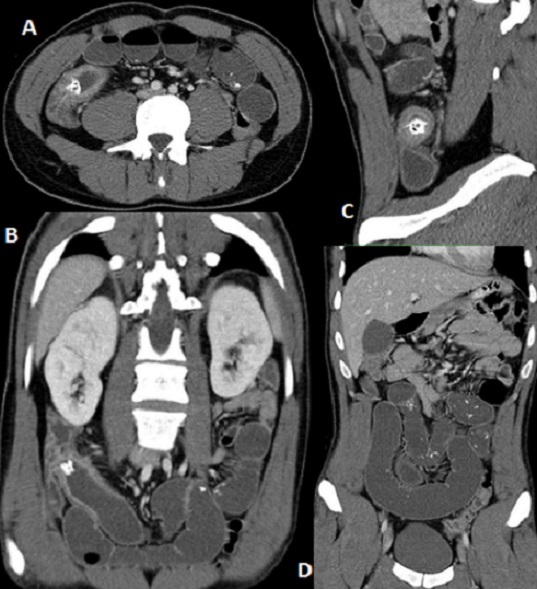
(A) coupe tomodensitométrique axiale passant par la fosse iliaque droite réalisée après injection intraveineuse de produit de contraste (temps portal): présence d'une image de densité calcique d'environ 17mm de diamètre (corps étranger), siégeant au niveau de la jonction iléo-cæcale, intra-luminale, en amont de la valvule de Bauhin; noter l’épaississement pariétal circonférentiel régulier bien rehaussé de la dernière anse iléale (réactionnelle), traduisant le caractère enclavé obstruant de ce corps étranger); (B) coupe tomodensitométrique coronale oblique passant par le plan de la symphyse pubienne réalisée après injection intraveineuse de produit de contraste (temps portal): meilleure illustration du caractère obstruant du corps étranger intra-luminal, la zone de transition et l'occlusion en amont; (C) coupe tomodensitométrique sagittale stricte passant par la jonction iléo-cæcale réalisée après injection intraveineuse de produit de contraste (temps portal): noter l’épaississement pariétal circonférentiel régulier bien rehaussé de la dernière anse iléale, traduisant le caractère enclavé obstruant de l'objet intra-luminal; (D) coupe tomodensitométrique coronale stricte passant par le plan de la symphyse pubienne réalisée après injection intraveineuse de produit de contraste (temps portal): illustration de la distension des anses iléales (occlusion en amont de l'obstacle décrit sur la figure A); les anses ont un contenu liquidien (stase) avec quelques images hyperdenses de même densité que l'obstacle iléo-cæcal

